# Our War Nurses

**Published:** 1920-10-16

**Authors:** 


### Our War Nurses.

While we hear a great deal, and rightly so, about the men who fought and suffered in the war, and who are still waiting to be placed in industry, we must not forget the nurses who served their country from 1914 onwards, in the majority of cases with a heroism scarcely less than that of the men themselves. At the present moment there are on the register of the Nurses' Resettlement Department, which is affiliated to the Professional Women's Register, at 99 Queen's Gate, S.W. 7, the names of over 550 fully-trained and partly-trained nurses who are seeking well-paid, and for the most part non-residential, professional employment. Most of these nurses have now had a much-needed rest after their prolonged war service, and they are anxious to undertake further work for the civil population in such appointments as surgical assistants (non-residential), dental assistants, visiting nurses, private masseuses, school and dental nurses, health inspectors, investigation officers, and probation officers.

The demand for these varied types of work is not limited to London. In the provinces nurses are registering their requirements at the Professional Women's Registers scattered over the country, and in Scotland at the offices at 112 George Street, Edinburgh.

As an example of the work of the Nurses' Resettlement Committee, it may be stated that out of a total of 6,150 individual inquiries 2,015 have been placed in satisfactory civil employment, whilst a further 3,594 have been put into touch with training.

The Nurses' Resettlement Department is in close touch with various Government Departments, such as the India Office, the Home Office, Ministry of Pensions, Ministry of Health, Metropolitan Asylums Board, and various overseas nursing associations and services, such as Lady Minto's Indian Nursing Service, Lady Muriel Paget's Mission to Russia, the League of Red Cross Societies, the Egyptian Government (Medical Department).

Owing to the dearth of administrative appointments in the nursing profession, it has been found that many well-trained, experienced nurses are seeking an outlet for their energies in work of another kind, such as poultry farming, gardening, tea shops, and club work.

